# A case of a misleading conclusion: a critical reassessment of the methodological and interpretive flaws of a recent meta-analysis of the vector role of cat fleas in feline hemotropic *Mycoplasma* species transmission

**DOI:** 10.1186/s13071-025-07143-w

**Published:** 2025-11-16

**Authors:** Mahmoud S. Safwat

**Affiliations:** Department of Internal Medicine and Infectious Diseases, Faculty of Veterinary Medicine, Cairo University, Giza, 12211 Egypt

**Keywords:** *Ctenocephalides felis*, Feline hemotropic *Mycoplasma* species, Fleas, Hemoplasmas, Meta-analysis, Transmission, Vector

## Abstract

**Abstract:**

A recent meta-analysis published in *Parasites & Vectors* [17(1):444, 2024] re-evaluated the prevalence of feline hemotropic *Mycoplasma* spp. in *Ctenocephalides felis*. The authors compared prevalence studies using different PCR primers: those employing Jensen or Manvell primers reported high prevalence (~ 33%), while others showed much lower rates (< 1%). To investigate potential Jensen/Manvell primer non-specificity, the authors used both primers and sequencing to reanalyze archived individual flea samples from pools previously reported as positive by Jensen primer-based PCR. Based on low prevalence and non-specific amplifications, they questioned primer specificity and concluded that prevalence was lower than previously reported, and that *C. felis* is less likely to be a vector of feline hemotropic *Mycoplasma* spp. This correspondence critically reassesses that conclusion by highlighting multiple methodological and interpretive flaws in the meta-analysis. Sequencing-confirmed *Mycoplasma* spp. detections from Jensen primer-based studies were disregarded and key data from source studies, including prevalence values and flea washing/pooling practices, were misreported in the meta-analysis. The reanalysis experiment suffered from design limitations, including mismatched sampling units (individual vs pools), unclear distribution of results across previously reported positive pools, and unaddressed confounders such as DNA degradation and contamination over ~ 12 years of storage. Grouping Jensen and Manvell primers, despite their distinct diagnostic behaviors, further undermines the analysis. Alternative explanations for prevalence variation, such as population heterogeneity, pooling flea samples, primer sensitivity, and strain variation, were not considered. The citations were used selectively to emphasize supporting studies, while contradictory evidence was omitted. Due to these limitations, the revised prevalence estimate is not supported, and the vectorial role of *C. felis* remains unresolved, necessitating further well-designed studies to establish its true epidemiological role.

**Graphical Abstract:**

**Figure:**
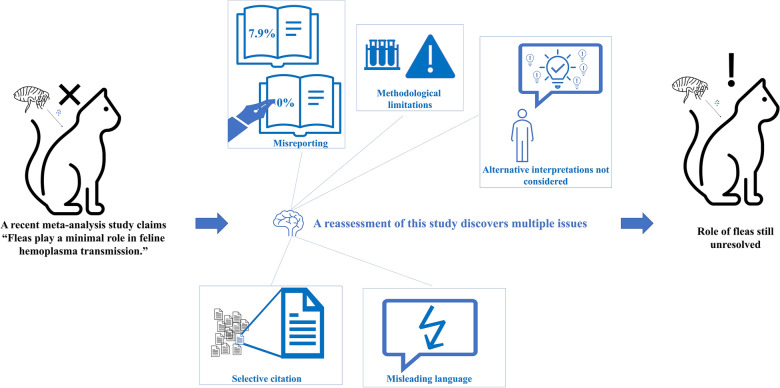

## Background

I read with interest the recent article titled “A case of mistaken identity: a systematic review, meta-analysis, and reinvestigation of hemotropic *Mycoplasma* spp. infection in* Ctenocephalides felis* (cat flea)” [1], which provides a meta-analysis that re-examines the prevalence of feline hemotropic *Mycoplasma* spp. in *C. felis*. Reports of high *Mycoplasma* spp. prevalence in *C. felis* have been considered key evidence supporting a potential vectorial role for *C. felis*. The meta-analysis indicated that previous research using primers designed by Jensen et al. [2] and Manvell et al. [3] yields substantially higher prevalence estimates (33%) than studies using other primers (< 1%). Specifically, seven studies used Jensen or Manvell primers (one used the Manvell primer [4] and six used the Jensen one [5–10]), while six studies used other primers [11–16]. The authors question the specificity of the Jensen/Manvell primers, suggesting that they may potentially influence prevalence estimates. The authors tested this hypothesis by reanalyzing archived flea samples. Based on the low prevalence and non-specific amplifications observed in their experiment, they concluded that *Mycoplasma* spp. prevalence in *C. felis* is lower than previously reported and suggested that *C. felis* could play a limited role in the transmission of these species.

The meta-analysis represents a timely and important contribution to an ongoing debate. However, upon close reassessment, several methodological and interpretive issues emerge that may affect the conclusions drawn from it. The aim of this correspondence is to clarify these concerns and provide alternative interpretations grounded in published data and sequencing-confirmed evidence, as outlined below.

## The misreporting of key data from source studies

### Studies using Jensen/Manvell primers

Although it was acknowledged that three studies using Jensen/Manvell primers included sequencing data [4–6], these were not considered in the interpretation of the meta-analysis. Two of these studies [5, 6], both using the Jensen primer, confirmed *Mycoplasma* spp. identity through sequencing in all positive samples, which is inconsistent with the hypothesis of widespread non-specific amplification. The third study [4], using the Manvell primer, reported no positives and is therefore neutral in this context.

The meta-analysis also indicated that the study by Shaw et al. [7], which used the Jensen primer, neither washed fleas nor sequenced the PCR positives (Supplementary File 2 of the article). In fact, both washing and sequencing were performed, with sequencing confirming *Mycoplasma* spp. in all sequenced samples [7]. This distinction is important, as Shaw et al. [7] is the only Jensen primer-based study that washed fleas before testing. By not accurately reporting these methodological details, the meta-analysis may inadvertently underrepresent evidence relevant to the potential vectorial role of *C. felis*. Additionally, Table 3, which categorized studies included in the meta-analysis by primer use and flea washing, should be reconsidered in light of these corrections.

### Studies using other primers

The meta-analysis appears to misreport data from two studies [11, 12] in ways that may underestimate the prevalence of *Mycoplasma* spp. Zerea et al. [11], an Asian study, is listed as reporting 0% prevalence (Table 2; Supplementary File 2), whereas the original article documented 7/91 positives (7.9%). For the UK study by Abdullah et al. [12], the meta-analysis (Table 2; Supplementary File 2) indicates that 470 flea pools were examined, with 3/470 positives. While 470 pools were indeed tested, these included pools from all flea species, host species, or unidentified sources. According to the inclusion criteria of the meta-analysis, only *C. felis* collected from cats should have been considered. However, only 210 cats with *C. felis* were included. Using 470 instead of 210 as the denominator may have underestimated prevalence and inadvertently reinforced the authors’ hypothesis. When the data from these studies are corrected, the prevalence of *Mycoplasma* spp. in *C. felis* determined using non-Jensen primers increases from 8/949 (0.82%) to 15/689 (2.2%).

In addition, the flea samples in at least two studies used for the meta-analysis are inaccurately described as pooled when most of the fleas were tested individually. This distinction is important because individual testing naturally yields lower detection rates than pooled testing; presenting these results as pooled data may have contributed to the apparent misreporting of prevalence.

For the Swiss study by Willi et al. [13], it is reported that pools were examined for the meta-analysis, whereas the original study states that most fleas were tested individually. For the UK study by Abdullah et al. [12], 470 pools are reported for the meta-analysis, each containing 1–89 fleas. However, in the original study, the median number of fleas per pool was 1, with only a single cat having 89 fleas. At least half of the samples therefore comprised individual fleas, not pools.

## Methodological shortcomings

### Reanalysis of the Thai samples

Stored individual flea samples that previously tested positive by Jensen primer-based PCR without sequencing in the original Thai study [10] were reanalyzed for the meta-analysis, with the aim of evaluating primer specificity and its impact on reported *Mycoplasma* spp. prevalence. In this reanalysis, both PCR and sequencing were applied, and the observed prevalence was lower (2/67, 3% of individuals) than in the original study (17/50, 34% of pools). *Spiroplasma* spp. were also amplified in 18/67 samples, while the remaining 47 were negative.

While this reanalysis provides additional data, there are several methodological considerations that may affect its interpretation. The samples had been stored for over a decade, potentially influencing DNA integrity. Additionally, comparisons were made between individual fleas in the reanalysis and pooled fleas in the original study, which are not directly comparable and substantially influence observed prevalence, as noted by Willi et al. [13]. Moreover, even if the reanalyzed fleas came from originally positive pools, this does not imply that each individual flea was infected; a single infected flea would have been sufficient to render an entire pool positive. By overlooking these fundamental differences in sampling design, the authors made inherently invalid comparisons.

They also report in Table 2 that the original pools contained from one to three fleas each, which is mathematically inconsistent. The original Thai study [10] described collecting 226 fleas from 50 cats, with no indication that any were excluded, and did not specify a maximum pool size. It is claimed that 67 reanalyzed fleas originated from previously PCR-positive pools, but a maximum of three fleas per pool across 17 positive pools would yield 51 fleas, not 67. Furthermore, the distribution of the two *Mycoplasma*-positive, 18 *Spiroplasma*-positive, and 47 negative fleas across the original pools is not reported, leaving a gap in data interpretation.

### Inappropriate grouping of Manvell and Jensen primers into one category

Although the Manvell primer is almost homologous to the Jensen primer, there is evidence that they behave differently in flea studies and should not be treated as equivalent. The Jensen primer has consistently amplified flea-derived *Mycoplasma* spp. DNA, with sequencing confirmation across multiple studies [5–7]. In contrast, the Manvell primer did not amplify *Mycoplasma* spp. in fleas in either a previous flea study [4] or in the experiments for the meta-analysis (reanalysis of Thai samples and washing experiments). In the reanalysis experiment, the single Jensen-positive flea tested negative with the Manvell primer, which also produced more non-specific results than the Jensen one (Table 4). While validated in cats [3], the Manvell primer may preferentially amplify *Spiroplasma* spp. in flea samples. Grouping these primers conflates their distinctive diagnostic behaviors and may lead to the unintentional underrepresentation of reliable Jensen primer-derived results.

### Washing experiment

The authors studied the effect of flea washing on *Mycoplasma* spp. prevalence using 20 pools of five fleas each (10 washed, 10 unwashed). The flea pools were tested by utilizing Manvell primer-based PCR and sequencing; all pools tested negative for *Mycoplasma* spp., while 12 were positive for *Spiroplasma* spp. Based on these results, the authors concluded that washing does not influence *Mycoplasma* spp. detection. 

However, this experiment was inherently constrained because the infection status of naturally collected fleas was unknown, making it inappropriate to conclude from uniformly negative results that washing had no effect. Moreover, the experiment was conducted on pooled rather than individual fleas, which affects the probability of detecting positives.

Similar experiments need to be designed for future studies that use fleas potentially infected with *Mycoplasma* spp., for example, through a controlled feeding approach in which fleas are allowed to feed on experimentally infected cats by using flea chambers, as previously described [19].

## More plausible confounders and alternative interpretations

### Reanalysis of the Thai samples

The authors used the reanalysis of the Thai samples to suggest that the high prevalence originally reported was due to non-specific amplifications, and concluded that *Mycoplasma* spp. prevalence was lower than previously reported. However, alternative interpretations of the results were not given. Several factors could account for the lower prevalence that they observed, including long-term sample storage with potential DNA degradation, the use of individual fleas versus that of pooled fleas in the original study, possible contamination with *Spiroplasma* spp. during storage, and co-infections in which high *Spiroplasma* loads may have outcompeted *Mycoplasma* during sequencing (particularly as genus-specific PCR and/or deep sequencing were not employed). These factors limit the strength of the authors’ interpretation. Notably, some of the authors previously acknowledged long-term storage as a critical limitation, observing that originally positive samples tested negative over time [17].

### Significantly high prevalence in studies using the Jensen primer

The authors attributed the high prevalences reported in studies using the Jensen primer to non-specific amplification. However, when viewed in context, several more plausible explanations emerge for this. The first of these is population heterogeneity, as four of the six Jensen primer-based studies sampled cats from shelters or sanctuaries [6–9], where prevalence is plausibly relatively high, compared with only one of the six studies using other primers [14]. The second explanation concerns pooling strategy, as most of the Jensen primer-based studies analyzed flea pools, whereas most of the studies employing non-Jensen primers tested individual fleas, which naturally yield lower detection rates than pooled testing [13]. Thirdly, concerning primer performance, those used in non-Jensen primer-based studies may have been less sensitive to flea-derived DNA compared to the Jensen primer. Fourthly, geographic variation needs to be considered, as differences in prevalence may reflect regional variation [13]. Lastly, there may have been strain-level differences, for example, strains of *Mycoplasma* spp. may differ in their flea adaptability, which is analogous to the strain-specific virulence suggested by Tasker et al. [18].

## Misrepresentation of studies cited to justify the authors’ hypothesis

In their discussion, the authors selectively cite studies or specific data that appear to support their argument against a vector role for fleas, while omitting the limitations of these studies and disregarding literature with opposing findings. This selective use of citations exaggerates their argument and misrepresents the overall body of evidence.

### Experimental evidence(Article discussion section: point 1)

The only experimental study [19] that directly addressed flea transmission had several limitations, including a small sample size, potential variation in the immune status of cats, use of laboratory-adapted strains of *Mycoplasma* spp. and fleas, and the rearing of fleas under suboptimal conditions. While these limitations were clearly acknowledged in the original study, only the observed failure of transmission was reported from the meta-analysis, whereas critical context that would qualify this interpretation was omitted.

### Geographical evidence (Article discussion section: point 2)

Two large-scale studies, from Italy and Japan (*n* > 950 cats), are cited to argue against a geographical association between *Mycoplasma* infection and *C. felis*. However, the article does not mention a comparable large-scale Swiss study that found a significant association between infection prevalence and warmer regions [20].

## Misleading use of language

The authors’ presentation of the results of the reanalysis (2/67 *Mycoplasma*-positive, 18/67 non-specific, and 47/67 negative) is misleading throughout the manuscript. In the abstract and results, they emphasize the low prevalence of *Mycoplasma* spp. (3%) and compare it to the originally reported prevalence of 34% without clearly stating that the original figure was based on pooled flea samples and thereby accentuating the apparent drop in detection. (In the abstract they state that: “67 C. felis samples [34% previously reported hemoplasma positive] were subject to PCR and sequencing. By this method, hemoplasma was detected in only 3% of samples.” In the results they state that: “In total, only two C. felis [2/67, 3% vs. the previously published 34%]...”)

In contrast, when describing non-specific bacterial amplification they do not report the exact proportion, but use vague language to describe it. In the abstract, they state that: “In the remaining ‘hemoplasma-infected’ fleas, PCR amplified* Spiroplasma* or other bacteria...” In the results, they state that: “*Spiroplasma* spp. DNA was amplified and sequenced from a majority of previously PCR-positive samples...” Also mentioned repeatedly in the discussion are “frequent amplification,” “are frequently amplified,” and “the frequency of off-target amplification,” despite only 18/67 fleas testing positive for non-specific amplification. Given that non-specific amplifications were not reported in the studies sequencing Jensen-positive samples, and considering the methodological limitations of this reanalysis, such phrasing may inadvertently guide readers to a predetermined conclusion regarding primer specificity.

## Conclusion: an unjustifiably revised prevalence

The authors disregarded previously confirmed high prevalences determined by sequencing in several Jensen primer-based studies [5–7] and relied instead on a reanalysis experiment with methodological limitations to justify excluding all Jensen primer-derived data. They then recalculated prevalence using only data from non-Jensen primer-based studies, reporting it as < 1%. However, even this revised figure is approximately three times lower than the corrected value of 2.2%. Presenting this recalculation at the end of the discussion may unintentionally bias the reader’s interpretation and contribute unnecessary confusion to an already debated topic. The vectorial role of fleas in the transmission of hemotropic *Mycoplasma* species remains unresolved, and further rigorous, well-designed studies are required to clarify this issue.

## Data Availability

Data supporting the main conclusions of this study are included in the manuscript.
